# Transdiagnostic neuroanatomical risk in schizophrenia: integrating regional vulnerability indices with anthropometric and fitness-based markers of cardiometabolic health

**DOI:** 10.1016/j.nicl.2025.103897

**Published:** 2025-10-27

**Authors:** Hannah Rößler, Lara Hamzehpour, Oliver Grimm

**Affiliations:** a Goethe University Frankfurt, University Hospital, Department of Psychiatry, Psychosomatic Medicine and Psychotherapy, Heinrich-Hoffmann-Straße 10, 60528 Frankfurt am Main, Germany; b Goethe University Frankfurt, Faculty 15 Biological Sciences, Frankfurt am Main, Germany

**Keywords:** Schizophrenia, Comorbidities, Vulnerability, Neuroimaging, Fitness, Biomarker

## Abstract

Schizophrenia is a multisystem disorder affecting both brain and body, with patients exhibiting substantial somatic comorbidities including obesity, reduced physical fitness, and elevated cardiometabolic risk. While neuroimaging-derived Regional Vulnerability Indices (RVIs) have quantified brain structural deviations from disorder-specific patterns, their relationship to physical health in schizophrenia remains largely unexplored. In this study, we combined RVIs with detailed anthropometric and fitness assessments in 42 schizophrenia patients and 43 matched healthy controls. Participants underwent MRI-based cortical thickness analyses to derive RVIs for nine psychiatric, neurological, and metabolic disorders, alongside measurements of body composition, cardiorespiratory fitness, handgrip strength, and jump performance. Patients exhibited significantly higher RVIs for schizophrenia (Cohen’s D = −1.1), bipolar disorder (Cohen’s D = −0.9), Alzheimer’s (Cohen’s D = −0.6) and Parkinson’s disease (Cohen’s D = −0.7), and type 2 diabetes (Cohen’s D = −0.9). These overlapping neuroanatomical vulnerabilities across psychiatric, neurodegenerative, and metabolic conditions might reflect shared underlying genetic and physiological mechanisms. Principal component analysis revealed three latent dimensions: (1) general risk factors, (2) physical fitness deficits, and (3) psychosis-spectrum vulnerability, with the latter two showing significant differences between groups. These findings highlight that metabolic impairment and reduced physical fitness in schizophrenia are not merely secondary phenomena but constitute distinct dimensions of systemic vulnerability. Overall, our results support a brain–body conceptualization of schizophrenia, suggesting that RVIs may serve as biomarkers to guide precision medicine interventions integrating neuroprotective strategies with lifestyle management. Future research should incorporate longitudinal and multimodal assessments to clarify causal relationships and optimize individualized treatment approaches.

## Highlights

• Regional Vulnerability Indices (RVI) reveal brain–body links in schizophrenia.
• Patients show higher RVIs for psychiatric and neurodegenerative disorders.
• Patients show higher RVIs for type 2 diabetes.
• Latent dimensions link neuropsychiatric, metabolic, and fitness impairments.
• Findings support precision medicine integrating brain and metabolic health.

## 1 Introduction

Schizophrenia is a complex, heterogeneous disorder that affects both brain and body. Beyond delusions, hallucinations, and cognitive impairment, patients frequently show substantial somatic comorbidities such as reduced physical fitness, increased body fat, and central adiposity (Hamzehpour et al., 2023, Martinelli et al., 2025). These physical health deficits contribute to reduced life expectancy and quality of life (Laursen et al., 2012), highlighting schizophrenia not only as a psychiatric condition but as a multisystem disorder. Lifestyle factors such as poor diet, physical inactivity, and sedentary behavior, combined with socioeconomic disadvantage, medication effects, and illness-related neurobiology itself, further exacerbate these problems (Thakore et al., 2002, Thakore, 2005, Hamzehpour et al., 2024). Consequently, patients face a markedly increased risk for cardiometabolic disease, including a 2.15-fold higher incidence of Type 2 Diabetes (T2D) and metabolic syndrome rates of up to 58 %, compared with age-matched healthy controls (Dong et al., 2024, Vancampfort et al., 2015), emphasizing the importance of monitoring physical health in schizophrenia.

Anthropometric measures such as Body Mass Index (BMI), Waist-to-hip ratio (WHR), and body fat percentage, together with indicators of cardiorespiratory fitness (VO_2_max), are well-established predictors of cardiometabolic risk in schizophrenia (Vancampfort et al., 2017, Strassnig et al., 2011, Strassnig et al., 2014). Specifically, higher BMI and WHR have been shown to correlate with higher blood pressure and impaired glucose metabolism in patients with schizophrenia, while lower VO_2_max values are associated with increased premature mortality rates due to cardiovascular conditions (Henderson et al., 2009, Smith et al., 2020, Ding et al., 2024). However, their integration with neurobiological markers of disease vulnerability has been limited.

In parallel, advances in neuroimaging have enabled the derivation of Regional Vulnerability Indices (RVIs), which quantify individual deviations from disorder-specific brain structural patterns based on large-scale *meta*-analytic datasets such as those from the ENIGMA consortium (Enhancing Neuro Imaging Genetics through Meta-Analysis) (Kochunov et al., 2022). RVI approaches have indicated overlapping brain structural signatures across schizophrenia, Bipolar Disorder (BD), Major Depressive Disorder (MDD) and neurodegenerative diseases such as Alzheimer’s Disease (AD) and Parkinson’s Disease (PD) (Kochunov et al., 2021, Kochunov et al., 2022), consistent with shared genetic, physiological and morphological features (Hagenaars et al., 2016, Elliott et al., 2018, Yamada et al., 2019). Since many psychiatric disorders not only display substantial genetic correlations and exhibit overlapping symptomatology but also share common physical comorbidities such as weight gain, it becomes especially relevant that RVI analyses systematically compare neuroanatomical patterns across different psychiatric conditions (Gurholt et al., 2021). Yet the relationship between these brain-based vulnerability profiles and somatic health markers remains largely unexplored. While large-scale neuroimaging initiatives such as the UK Biobank have demonstrated the utility of RVIs in population samples, few studies have combined these brain-based vulnerability indices with detailed anthropometric and fitness assessments or calculated RVIs of somatic conditions in clinical schizophrenia cohorts, limiting translational insights into individualized health interventions.

This study aims to address this gap by combining RVIs with detailed physical fitness and anthropometric data in patients with schizophrenia (SZs) and healthy controls (HCs). By examining RVIs not only for schizophrenia but also for other psychiatric, neurological and cardio-metabolic disorders, we aim to determine whether brain–body vulnerability reflects transdiagnostic processes rather than being specific to schizophrenia alone. We formulated the following three hypotheses:

RVI patterns in schizophrenia overlap with those of other psychiatric and neurological disorders, reflecting shared biological architecture.
RVI patterns also exhibit similarity to somatic conditions such as T2D and high blood pressure, indicating links between brain structural vulnerability and systemic health.
Principal Component Analysis (PCA) of RVIs and physical health measures will reveal latent dimensions differentiating SZs from HCs.

By integrating neuroanatomical vulnerability indices with anthropometric and fitness measures, this study provides a multimodal perspective on schizophrenia as a disorder of both brain and body and highlights potential pathways for precision medicine interventions targeting both domains.

## 2 Methods

### 2.1 Participants

For this study 53 SZs were matched with 47 HC as to age and sex. SZs were mainly recruited from the Department of Psychiatry, Psychosomatic Medicine and Psychotherapy of the university clinic in Frankfurt am Main, Germany and surrounding psychiatric institutes via online postings and flyers. The psychiatrists in charge of the patients’ treatment established psychiatric diagnoses based on DSM-V criteria. The following requirements had to be fulfilled by both groups to be included: 1) no intake of benzodiazepines within the last two weeks; 2) no physical impairments that would hinder the performance in the fitness test; 3) no history of neurological disorders; 4) no current alcohol or drug abuse; 5) no Magnetic Resonance Imaging (MRI) contraindications. Additional criteria for the SZs were 1) diagnosis of schizophrenia and 2) history of stable medication for at least four weeks. All participants provided written consent to the study protocol, approved by the institutional review board of the Goethe University Frankfurt. The study was registered at the German clinical study register (www.drks.de) with the number DRKS00023907 and is additionally indexed at the WHO study register.

During quality control of the MRI data, 15 participants had to be excluded due to motion artifacts, resulting in n = 42 SZs and n = 43 HCs retained for further analysis.

### 2.2 Psychopathology assessment

Several established questionnaires were used to assess psychopathology in both SZs and HCs: Chapman Scale for Physical and Social Anhedonia (PAS, SAS), World Health Organization Disability Assessment Schedule (WHODAS), Calgary Depression Scale for Schizophrenia (CDSS).

Before performing tests to compare both groups as to psychopathology, we tested for normality within each group using the Shapiro-Wilk test. Parametric analyses were conducted using independent samples t-tests and non-parametric data were analyzed using the Mann-Whitney *U* test. Sample characteristics and group comparisons are summarized in Table 1.

**Table 1:** Study sample characteristics.

Characteristics	SZ (n = 42)	SD	HC (n = 43)	SD
males	29		29	
females	13		14	
age (years)	34.2	10.3	34.8	11.5
CPZeq	479	347		
PAS	12.2*	7.5	8.7	5.4
SAS	12.4**	7	8.8	6.5
WHODAS	34.4**	16.8	9.9	10.9
CDSS	4.6**	3.4	1.5	2.7
VO_2_max	35.3**	10.2	40.4	9.2
WHR	0.9**	0.1	0.8	0.1
WHtR	0.5**	0.1	0.5	0.1
BMI	27.2**	4.7	24.9	4.4
total body fat [%]	30*	7.7	26.4	8
skin folds sum	176.3**	52.9	137.4	57
jump width [cm]	150.7*	34	167.3	48.1
handgrip strength [kg]	34.6*	9.5	39.9	10

Note: SZ, schizophrenia patients; HC, healthy controls; SD, standard deviation; CPZeq, chlorpromazine equivalent; PAS/SAS, Physical and Social Anhedonia Scales; WHODAS, World Health Organization Disability Assessment Schedule; CDSS, Calgary Depression Scale for Schizophrenia; VO_2_max, maximal oxygen capacity; WHR, waist-to-hip ratio; WHtR, waist-to-height ratio. **p ≤ 0.01; *p ≤ 0.05.

### 2.3 Physical fitness test

All participants completed a fitness assessment consisting of body measurements (WHR; Waist-to-height ratio, WHtR; BMI; total body fat percentage) and physical exercise tests (VO_2_max, jump width, handgrip strength). Body fat was determined using a skinfold caliper on seven anatomical points (subscapular, biceps, triceps, abdominal, suprailiac, thigh, calf). The skin was pinched between index finger and thumb, then the caliper was applied about 1 cm from the fingers. Skinfolds were measured twice bilaterally and averaged. Body density was estimated using the age-specific Durnin and Womersley (1973) formula (biceps, triceps, suprailiac, subscapular), and body fat percentage derived via Brožek et al. (2006). The sum of all seven skinfolds served as an additional obesity marker (skin folds sum).

As part of the fitness evaluation a Takei dynamometer (Takei Analogue Hand Grips Dynamo Meter, PS219A) was used to test handgrip strength in kilograms. Participants were instructed to hold the dynamometer in one hand and squeeze it as tightly as possible while keeping their arm straight, hanging at their side (average of two trials per side). Lower body strength was quantified by performing a standing long jump test, where participants were required to stand at a marker, then jump simultaneously with both legs and land on both feet (best of two trials). The width of the jump was measured in centimeters from the marker to the back of the heel. The next part of the test battery was the Chester step test, determining the participants’ VO_2_max. Subjects were instructed to step on a 30 cm-step using one foot at a time, without jumping. A metronome was used to set the pace (five 2‑min levels increasing from 60 bpm by 20 bpm per stage). Heart rate was monitored before and during the test via a pulse band placed below the chest. The test ended when 80 % of the age‑predicted maximum (220-age) was reached or upon voluntary exhaustion. VO_2_max (mlO_2_/kg/min) was estimated when at least three levels were completed by plotting each level’s heart rates on a graphical datasheet and identifying the crossing of the maximum heart rate.

Data normality was tested with the Shapiro-Wilk test. Between‑group differences were analyzed with independent t‑tests or Mann-Whitney U tests, as appropriate. The fitness test results and group differences are summarized in Table 1.

### 2.4 MRI data acquisition and analysis

Structural MRI data were acquired on a 3 Tesla MRI scanner (Siemens Magnetom Prisma) at the Brain Imaging Center, Goethe University Frankfurt, using a 64-channel head coil. Anatomical scans were performed with a T1-weighted 3D MPRAGE sequence (TR = 2000 ms, TE = 2.12 ms, FoV = 256, 176 sagittal slices, voxel size = 1 × 1 × 1mm, flip angle = 8°). To minimize movement, participants’ heads were stabilized with a pillow, and ear plugs were provided.

Anatomical scans were processed with FreeSurfer (version6, recon-all) to obtain measurements for cortical thickness and surface area. FreeSurfer segmented 34 cortical regions per hemisphere as well as total surface area and mean thickness for both hemispheres on the Desikan-Killiany atlas (Desikan et al., 2006).

For quality control (QC), we partially followed the *ENIGMA Cortical Quality Control Protocol 2.0* (https://enigma.ini.usc.edu/). Cortical output from FreeSurfer was extracted and screened for outliers using ENIGMA scripts. Instead of generating MATLAB images as in the standard protocol, visual inspection of each subject was performed using Freeview, allowing direct correction of errors using recon-edit and control points. Special attention was paid to subjects flagged as outliers in multiple regions. FreeSurfer was re-run after editing. Data quality was classified on an ordinal scale based on the extent of manual editing and whether errors occurred only in common areas (e.g., inferior frontal lobe, temporal pole) or in additional regions. During QC, 15 participants (5 HC, 10 SZ) were excluded due to insufficient FreeSurfer output quality, mostly caused by motion artifacts.

### 2.5 RVI analysis

RVIs constitute a neuroimaging-derived metric that quantifies the similarity between an individual’s structural brain profile and *meta*-analytically defined disorder-specific expected patterns (EPs). The RVI was calculated based on the FreeSurfer-output for cortical thickness, using the RVIpkg package in R as the mean cortical thickness across hemispheres for each corresponding FreeSurfer-region. To control for potential confounding effects linear regression was used, including age, sex, intracranial volume (ICV), comorbidities (Attention Deficit/Hyperactivity Disorder, T2D) and ordinal scale for image quality (see section 2.4) as covariates. Subsequently, residuals were extracted, followed by an inverse normal transformation (INT) which was performed on these residuals based on their ranks. The INT-transformed values were then z-normalized using the mean and standard deviation of the full study sample. The RVI was then computed for each subject as the Pearson correlation coefficient between the z-normalized INT residuals and the disorder-specific EP. The index was calculated for the following nine disease classes: schizophrenia spectrum disorder (SSD), BD, MDD, AD, PD, hypertension (highBP), metabolic syndrome (T2D, highBP, hyperlipidemia), T2D, Attention Deficit/Hyperactivity Disorder (ADHD). The ADHD-EP effect sizes were derived from Hoogman et al. (2019; Supplementary Table ST13) and AD-EP derived from Kochunov et al. (2021). All other EPs were available within the RVIpkg package.

For each disorder-specific RVI, group differences between HCs and SZs were assessed using independent two-sample t-tests. To account for multiple testing, the False discovery rate (FDR) correction was applied.

To assess the sensitivity of the group comparisons, we conducted post-hoc power analysis. Power was estimated for the observed effect sizes (Cohen’s D) as well as for small (d = 0.2), medium (d = 0.5) and large (d = 0.8) effects.

### 2.6 PCA

Finally, we performed a PCA to discover latent factors of brain-body vulnerability while reducing dimensionality. All RVIs, psychopathological (PAS, SAS, WHODAS, CDSS), and fitness (VO_2_max, WHR, WHtR, BMI, total body fat percentage, skinfold sum, jump width, handgrip strength) data were included. The Kaiser-Meyer-Olkin (KMO) test assessed dataset suitability, yielding an initial overall measure of sampling adequacy (MSA) of 0.6, with individual MSA values calculated for each variable (SAS = 0.47, PAS 0.6, VO_2_max = 0.78, BMI = 0.48, WHR = 0.36, WHtR = 0.51, skin folds sum = 0.78, total body fat = 0.7, jump width = 0.84, hand grip strength = 0.55, WHODAS = 0.63, CDSS = 0.58, AD-RVI = 0.44, ADHD-RVI = 0.56, T2D-RVI = 0.76, MDD-RVI = 0.75, high BP-RVI = 0.58, metabolic syndrome-RVI = 0.69, BD-RVI = 0.49, SSD-RVI = 0.48, PD-RVI = 0.77). Although the general recommendation is to exclude variables with MSA below 0.5 to improve model fit, the RVIs for Schizophrenia and BD, which had values below this threshold, were retained due to their theoretical relevance to core constructs (e.g., “psychosis-spectrum vulnerability”) and consistent high loadings in preliminary analyses, prioritizing clinical and theoretical interpretability (see Supplementary Material 3). Other variables with MSA below 0.5 (WHR, AD-RVI, SAS, BMI) were excluded, raising overall MSA to 0.7.

PCA was conducted using the principal() function in RStudio, focusing on shared variance to address limited sample size and enhance stability, applying varimax rotation for interpretability. Parallel analysis indicated retention of the first three principal components.

Group comparisons between HCs and SZs used independent samples t-tests on scores of these components.

## 3 Results

### 3.1 Sample characteristics

Sample characteristics are summarized in Table 1.

Mann–Whitney U tests revealed significantly higher psychopathology scores in the SZs across all questionnaires compared to HCs (PAS, Mann–Whitney U = 629.5, p = 0.02; SAS, Mann–Whitney U = 591, p = 0.006; WHODAS, Mann–Whitney U = 180.5, p ≤ 0.0001; CDSS, Mann–Whitney U = 358, p ≤ 0.0001).

Anthropometric measures indicated also significantly lower health status in SZs compared to HCs (WHR, t = −3.18, p = 0.002; WHtR, t = −3.2, p = 0.002; BMI, Mann-Whitney U = 581, p = 0.004; total body fat percentage, t = −2.13, p = 0.04; skin folds sum, Mann-Whitney U = 522.5, p = 0.0008).

HCs performed significantly better across all disciplines of the fitness test battery including jump width (Mann-Whitney U = 1142; p = 0.04), VO_2_max (Mann-Whitney U = 1211, p = 0.007), and handgrip strength (Mann-Whitney U = 1168.5; p = 0.02).

### 3.2 RVI results

**Fig. 1:**
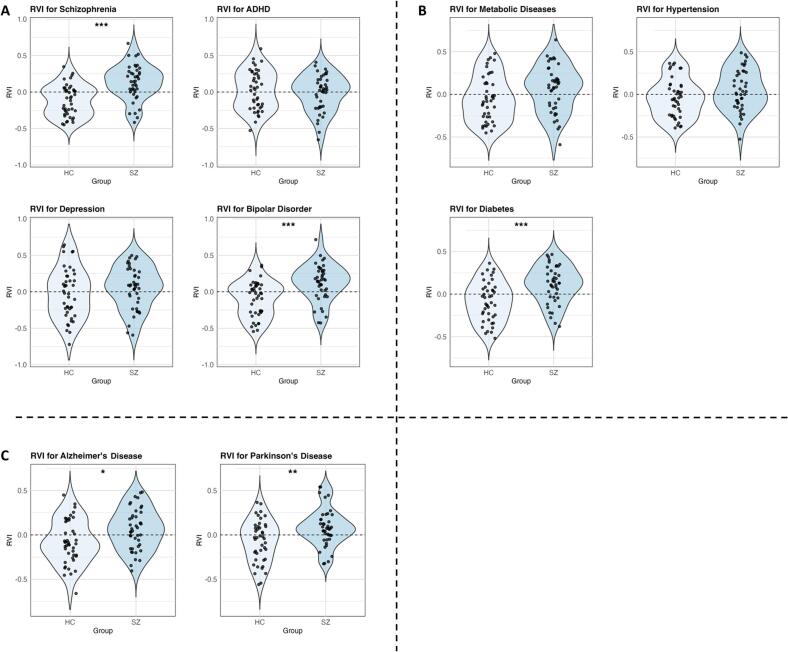
Violin plots of Regional Vulnerability Index (RVI) analysis. A depicts RVI scores for psychiatric conditions comparing patients with schizophrenia (SZ) and healthy controls (HC). B shows group comparisons of RVI scores for metabolic diseases. C displays group comparisons of RVI scores for neurodegenerative diseases. Abbreviations: ADHD = Attention Deficit/Hyperactivity Disorder; Diabetes = type 2 diabetes. *p ≤ 0.05; **p ≤ 0.01; ***p ≤ 0.001 (FDR-corr.).

The RVI reflects deviation in cortical thickness from disorder-specific patterns. Higher RVI values indicate greater similarity to disorder-specific EPs, thus reflecting increased neuroanatomical vulnerability. It was examined whether SZ exhibit a higher RVI for schizophrenia itself, as well as for other conditions. RVI results and group comparisons are summarized in the violin plots of Fig. 1. All p-values reported are adjusted for multiple comparisons using the False Discovery Rate (FDR) correction.

The schizophrenia-specific RVI showed significantly higher values in SZs compared to HCs (t = −4.99; adjusted p ≤ 0.0001; Cohen’s D = −1.1).

RVIs were also significantly elevated for BD (t = −4.09; adjusted p = 0.0004; Cohen’s D = −0.9), AD (t = −2.74; adjusted p = 0.01; Cohen’s D = −0.6), PD (t = −3.04; adjusted p = 0.007; Cohen’s D = −0.7) and T2D (t = −3.95; adjusted p = 0.0005; Cohen’s D = −0.9).

No significant differences between SZs and HCs were observed for the remaining RVIs: MDD (t = −1.05; adjusted p = 0.33), metabolic syndrome (t = −1.89; adjusted p = 0.09), high BP (t = −1.75; adjusted p = 0.11) and ADHD (t = 0.68; adjusted p = 0.5). Detailed post-hoc power analysis results for all comparisons are provided in Supplementary Table 1.

### 3.3 PCA results

The PCA revealed three principal components which explained a cumulative 64.62 % of the total variance. Among them, the second (t = −3.2; adjusted p = 0.003; Cohen’s d = −0.7) and third (t = −6.24; adjusted p ≤ 0.0001; Cohen’s d = −1.36) showed significant differences between HCs and SZs. FDR correction was applied to adjust for multiple comparisons. The effect observed in the first component did not reach significance (t = −0.7; adjusted p = 0.49).

**Fig. 2:**
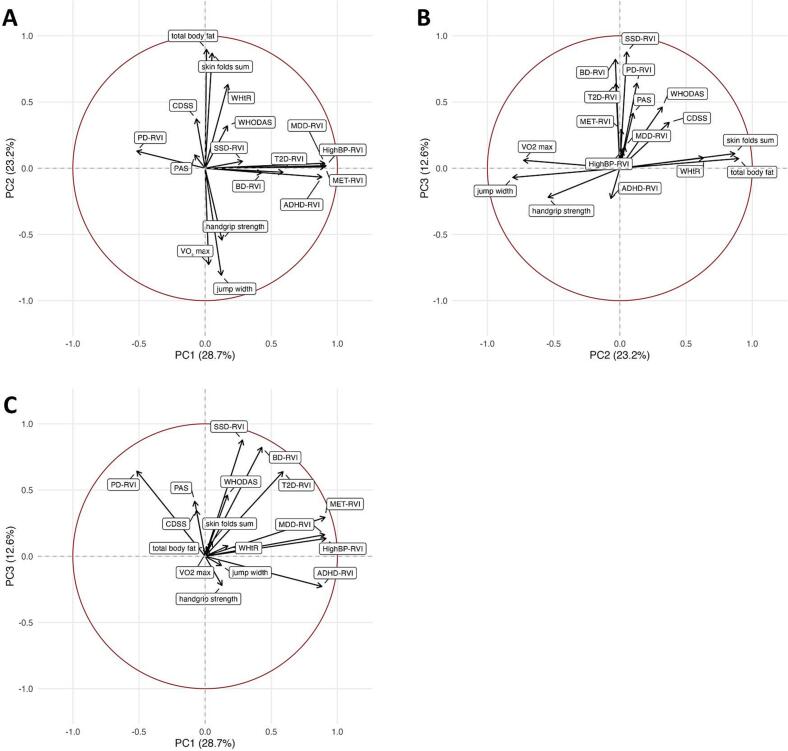
Biplots of Principal Component Analysis (PCA). Biplots display the variable loadings and observation scores for the first three principal components. Arrows indicate the direction and strength of each variable’s loading, with variables pointing in similar directions being positively correlated. Panels show PC1 vs. PC2 (A), PC2 vs. PC3 (B), and PC1 vs. PC3 (C), allowing visualization of how general risk variables, physical fitness measures, and psychosis-spectrum RVIs are distributed across the three independent components. Percentages in parentheses indicate the proportion of total variance explained by each principal component. Abbreviations: PC = Principal Component; RVI = Regional Vulnerability Index; WHtR = Waist-to-Height Ratio; WHODAS = WHO Disability Assessment Schedule; CDSS = Calgary Depression Scale for Schizophrenia; PAS = Physical Anhedonia Scale; BD = Bipolar Disorder; MDD = Major Depressive Disorder; PD = Parkinson’s Disease; ADHD = Attention-Deficit/Hyperactivity Disorder; MET = Metabolic Syndrome; DM = Type 2 Diabetes; HighBP = High Blood Pressure; SSD = Schizophrenia Spectrum Disorder.

**Fig. 3:**
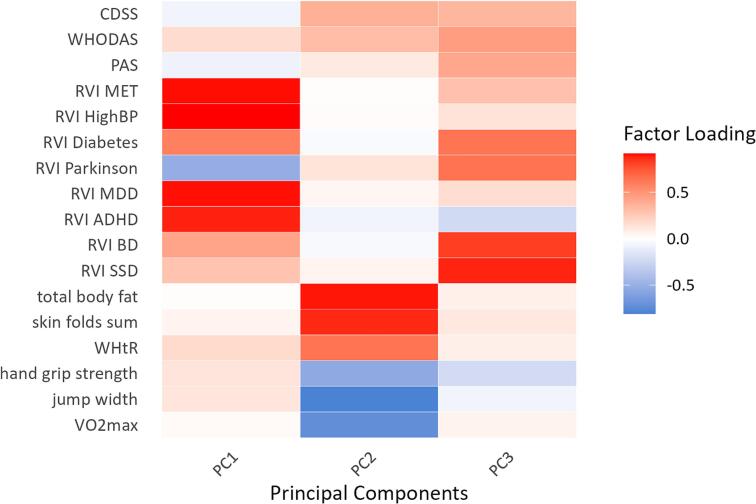
Factor loadings on three principal components after Principal Component Analysis (PCA). All variables included in the PCA are listed on the left side and grouped into different categories: psychopathology (CDSS, Calgary Depression Scale for Schizophrenia; WHODAS, World Health Organization Disability Assessment Schedule; PAS, Chapman Scale for Physical Anhedonia), regional vulnerability indices (RVI; MET, metabolic diseases; HighBP, high blood pressure; Diabetes, type 2 diabetes; Parkinson, Parkinson’s Disease; MDD, Major Depressive Disorder; ADHD, Attention Deficit/Hyperactivity Disorder; BD, Bipolar Disorder; SSD, Schizophrenia Spectrum Disorder), anthropometric measures (total body fat; skin fold sum; WHtR, waist-to-height ratio) and physical fitness variables (hand grip strength; jump width; VO2max, maximal oxygen capacity). The color bar on the right indicates high positive loadings as red and high negative loadings as blue. According to the loading pattern principal component 1 (PC1) was named ‘Neuropsychiatric and Vascular Risk Profile’, PC 2 was termed ‘Metabolic and Physical Fitness Dimensions’ and PC3 was called ‘Psychosis-spectrum Vulnerability’. (For interpretation of the references to colour in this figure legend, the reader is referred to the web version of this article.)

Fig. 2 presents the PCA biplots, enabling assessment of the contribution and mutual orientation of variables across the extracted components, while Fig. 3 depicts the factor loadings.

Based on the loading pattern, the first component was labeled “General Risk Profile” (PC1), as it reflects a composite of neurological, psychiatric and cardiovascular vulnerability. This component was predominantly characterized by high positive loadings for ADHD risk, high BP risk, MDD risk and metabolic syndrome risk factors. In contrast, the PD risk factor exhibited a negative loading (Fig. 3).

The second component, interpreted as “Physical Fitness Dimensions” (PC2), was mainly influenced by fitness-related and anthropometric measures. Positive loadings were observed for total body fat percentage, skin folds sum, and WHtR. Negative loadings were jump width, VO_2_max, and handgrip strength (Fig. 3).

The third component captures multiple indicators of psychiatric vulnerability and was therefore labeled “Psychosis-spectrum vulnerability” (PC3). High positive loadings were found for schizophrenia risk, BD risk, T2D risk, and PD risk (Fig. 3).

These labels are based on interpretations of dominant loading patterns and theoretical relevance and should thus be considered hypothesis-generating as well as subject to further validation in future studies.

As it can be taken from Fig. 2, PC1 and PC2 as well as PC2 and PC3 appear largely orthogonal, indicating that general risk, psychosis-spectrum vulnerability variables and physical fitness measures load on distinct dimensions. Fig. 2C shows that RVIs for schizophrenia, BD, PD, and T2D cluster in the same quadrant, reflecting shared positive loadings on both components.

Complementary sensitivity analyses using full factor analysis produced similar factor structures, supporting the robustness of the findings.

## 4 Discussion

This study is among the first to systematically integrate transdiagnostic, neuroanatomically derived RVIs with comprehensive physical health parameters obtained by anthropometric measures in SZs and HCs.

First of all, the results revealed significantly elevated RVI scores for SSD in SZs compared to HCs, validating the method’s sensitivity to detect known disorder-specific cortical patterns even in a relatively small sample.

Secondly, in line with the hypotheses, RVIs were significantly increased in SZs for BD, AD and PD. These results are consistent with previous studies that point out not only a genetic link between schizophrenia and BD, but also similarities on a physiological and morphological level. While both disorders share symptoms such as cognitive deficits, anhedonia, mood swings and even psychosis, both patient groups show ventricular enlargement and global brain volume reduction, as well as abnormal expression of proteins in hippocampal and prefrontal regions (Yamada et al., 2019). Furthermore, Wingo and colleagues (2022) summarized the shared genetic basis of psychiatric and neurodegenerative conditions in 13 proteins which represent about 30 % of all causal proteins responsible for neurodegeneration. In particular with regard to AD, PD, and schizophrenia Apolipoprotein E plays a central genetic role in mediating neurodegenerative and inflammatory pathways (Jonas et al., 2019, Liampas et al., 2024). Clinically, these disorders share symptoms such as psychosis and cognitive decline. Our findings corroborate these overlaps linked to neurodegeneration on a morphological level and − moreover − provide evidence that RVIs constitute a robust tool which can be used to identify neuroanatomical vulnerability across psychiatric and neurological conditions.

Despite documented genetic overlaps (Demontis et al., 2019, Cross-Disorder Group of the Psychiatric Genomics Consortium, 2019), we found no significant MDD-RVI and ADHD-RVI elevation. This aligns, however, with recent neuroimaging *meta*-analyses showing the lowest correlation between a shared cortical thickness pattern in MDD and schizophrenia (Cao et al., 2023). Furthermore, as ADHD cortical differences normalize with age while schizophrenia patterns persist (Zhang-James et al., 2021), our lack of significance may reflect age-dependent neuroanatomical trajectories.

Beyond the overlaps with other psychiatric and neurodegenerative conditions, our analyses revealed significantly elevated RVIs for T2D in SZs. Emerging research supports shared pathophysiological mechanisms between schizophrenia and T2D, encompassing genetic predispositions, chronic low-grade inflammation, immune dysregulation and impaired glucose metabolism (Arruda et al., 2024, Dong et al., 2024). These overlapping mechanisms likely affect brain structure and function, particularly in areas critical for cognitive and metabolic regulation, such as the hippocampus and prefrontal cortex, where insulin signaling influences neurogenesis and neuronal remodeling. Given that T2D is highly prevalent in schizophrenia, with epidemiological studies reporting up to a 3-fold increased risk, even suggesting schizophrenia as a risk factor for T2D (Mizuki et al., 2021, Dong et al., 2024), it is plausible that metabolic dysregulation contributes significantly to structural brain changes.

In contrast, although hypertension and metabolic syndrome frequently co-occur with schizophrenia, we did not observe significantly elevated RVIs for these conditions. One explanation may be that T2D manifests earlier and produces more lasting, direct neurotoxic effects via insulin resistance and hyperglycemia comprising brain integrity (Arruda et al., 2024). Contrarily, the metabolic syndrome RVI encompasses a heterogeneous group of conditions with diverse effects on the brain and other cardiometabolic components, such as hypertension, may exert more variable or later-stage effects that are less robustly captured by RVI metrics. However, a longitudinal study would be important to substantiate these assumptions. Nonetheless, our findings extend the concept of transdiagnostic brain vulnerability to major somatic conditions, highlighting that structural brain profiles in schizophrenia may reflect neuroanatomical patterns of cardiometabolic dysregulation. Studying the RVI–T2D relationship thus offers important insights into the brain–body interplay on this systemic disorder.

To integrate these multidimensional findings and identify latent patterns of brain-body vulnerability, we employed PCA. This approach allowed us to reduce the complexity of multiple RVIs and physical health measures into interpretable components, revealing how neuroanatomical vulnerability patterns cluster with transdiagnostic somatic health indicators (Fig. 2). The first component (“General Risk Profile”) showed high positive loadings for both neuropsychiatric conditions as well as cardiometabolic diseases such as T2D and hypertension. This component did not significantly differentiate SZs from HCs, suggesting that it may reflect a common clustering of interrelated risk factors that exist broadly across the general population rather than a disorder-specific signature. The second component (“Physical Fitness Dimensions”) yielded significantly higher scores in SZs, indicating higher levels of visceral body fat and reduced physical fitness, emphasizing that physical health impairments are not merely secondary observations but represent a different part of the disorder. The third component (“Psychosis-Spectrum Vulnerability”), too, revealed significant differences between groups. It showed strong positive loadings for RVIs of schizophrenia, BD, T2D and PD, reflecting a shared neuroanatomical signature across psychiatric and somatic conditions, likely rooted in overlapping mechanisms such as oxidative stress, neuroinflammation and insulin resistence (Wingo et al., 2022). The fact that the RVIs of hypertension and metabolic syndrome did not load on this component may suggest that insulin resistance represents the core latent process contributing to neurodegeneration and accelerated brain aging, which would be in line with the neurodegenerative hypothesis of schizophrenia (Stone et al., 2023).

The orthogonality of components 2 and 3 (Fig. 2) indicates that metabolic-physical and neuropsychiatric vulnerabilities represent independent dimensions of illness burden, in line with factor-analytic studies (Wallwork et al., 2012, Stein et al., 2020) showing that cognitive, positive, negative, and physical health features of schizophrenia load onto distinct factors. Clinically, this separation suggests potential for stratified interventions—for example, prioritizing metabolic and physical health in patients with high component 2 scores while focusing on neuroprotective strategies in those with elevated component 3 scores. RVIs could serve as biomarkers to guide and evaluate such tailored approaches. Importantly, although these domains dissociate statistically, both are significantly impaired in SZs, pointing to a brain–body integration in which cardiometabolic and neuropsychiatric risk profiles coexist yet vary independently at the individual level. Physical comorbidities remain a central feature of systemic vulnerability in schizophrenia. High rates of metabolic syndrome (Vancampfort et al., 2015) and reduced energy expenditure (Sharpe et al., 2006) signal substantial metabolic impairment, emphasizing lifestyle factors as part of an intrinsically influenced systemic risk profile exacerbated but not solely caused by treatment (Stahl et al., 2009). Shared genetic links connect schizophrenia with diet (Niarchou et al., 2020), cognitive ability, and physical health traits (Hagenaars et al., 2016), with inflammation and immune pathways likely bridging brain and cardiometabolic risk (So et al., 2019). Even healthy individuals with schizophrenia-like brain patterns show poorer cardiovascular/metabolic health and signs of brain aging (Ma et al., 2023), supporting a shared brain–body pathophysiology.

When interpreting these findings, several methodological considerations and limitations should be acknowledged. While the RVI offers a promising approach to quantify individual brain vulnerability patterns related to schizophrenia and associated comorbidities, the current implementation primarily focuses on cortical thickness measures, thereby excluding potentially relevant subcortical and white matter structures involved in metabolic regulation and brain–body interactions. Moreover, the EP against which individuals are compared is derived from heterogeneous source studies with varying population characteristics, which could introduce variability and limit generalizability. Apart from that, as with any correlational measure, RVIs do not allow us to distinguish between true vulnerability and compensatory or adaptive changes in brain structure. While RVIs were designed to reflect deviations from cortical volume (Kochunov et al., 2021), it remains unclear whether observed changes represent pathological loss or compensatory remodeling. Future longitudinal research should aim to clarify these biological interpretations. Clinical phenotyping and physical health measurements in this context also have limitations: objective metabolic assessments such as HbA1c, oral glucose tolerance tests, or direct blood pressure monitoring were not available, and reliance on anthropometric proxies may reduce measurement precision and obscure more nuanced metabolic dysfunction. In addition, the cross sectional design precludes conclusions about causality or temporal sequencing of brain and physical health changes. Although antipsychotic medication may exert similar effects on brain structure and metabolism that cannot be disentangled in a cross-sectional design (Tuominen et al., 2025), the elevated T2D-RVI parallels findings in medication-naïve patients (Rajkumar et al., 2017), suggesting at least partial independence from treatment. In our sample, a post-hoc analysis revealed no significant correlations between antipsychotic medication dose (CPZeq) and key RVI or PCA component scores (see Supplementary Table 2). Finally, as indicated by our post-hoc analysis (Supplementary Table 1), the relatively small sample size may limit power to detect weaker effects (e.g. hypertension RVI, MDD RVI, ADHD RVI), a common issue in neuroimaging research (Marek et al., 2022), highlighting the need for replication in larger, independent cohorts. Thus, non-significant findings may reflect limited statistical sensitivity rather than the absence of true group differences. Nevertheless, moderately sized samples can still yield valuable insights when combined with multivariate, well validated models, supporting the methodological justification and careful interpretation of the present study.

Future research should integrate biochemical markers and comprehensive metabolic profiling with RVI measures, and extend analyses beyond cortical thickness, including subcortical and white matter structures. Longitudinal designs will be essential to clarify causal brain–body relationships and track changes over time.

In sum, these findings support viewing schizophrenia as a multisystem disorder. RVIs offer potential for precision medicine approaches serving as clinically useful biomarkers to identify individuals at high risk for somatic comorbidities − especially when brain and physical health assessments are combined to guide targeted, individualized therapy such as lifestyle interventions (Walburg et al., 2023).

## Role of the funding source

The study was supported by the 10.13039/501100001659German Research Foundation (DFG; grant number: 445498183), and the DYNAMIC center, funded by the LOEWE program of the Hessian Ministry of Science and Arts (grant number: 1/16/519/03/09.001(0009)/98).

## CRediT authorship contribution statement

**Hannah Rößler:** Writing – original draft, Visualization, Methodology, Formal analysis, Data curation. **Lara Hamzehpour:** Writing – review & editing, Writing – original draft, Visualization, Investigation, Data curation. **Oliver Grimm:** Writing – review & editing, Validation, Supervision, Project administration, Funding acquisition, Conceptualization.

## Declaration of competing interest

The authors declare that they have no known competing financial interests or personal relationships that could have appeared to influence the work reported in this paper.

## Data Availability

The authors do not have permission to share data.

## References

[b0005] Arruda A.L., Khandaker G.M., Morris A.P., Smith G.D., Huckins L.M., Zeggini E. (2024). Genomic insights into the comorbidity between type 2 diabetes and schizophrenia. Schizophrenia.

[b0010] Brožek J., Grande F., Anderson J.T., Keys A. (2006). Densitometric analysis of body composition. revision of some quantitative assumptions. Ann. N. Y. Acad. Sci..

[b0015] Cao Z., Cupertino R.B., Ottino-Gonzalez J., Murphy A., Pancholi D., Juliano A., Chaarani B., Albaugh M., Yuan D., Schwab N., Stafford J., Goudriaan A.E., Hutchison K., Li C.S.R., Luijten M., Groefsema M., Momenan R., Schmaal L., Sinha R., Garavan H. (2023). Cortical profiles of numerous psychiatric disorders and normal development share a common pattern. Mol. Psychiatry.

[b0020] Cross-Disorder Group of the Psychiatric Genomics Consortium, Cross-Disorder Group of the Psychiatric Genomics Consortium (2019). Genomic relationships, novel loci, and pleiotropic mechanisms across eight psychiatric disorders. Cell.

[b0025] Demontis D., Walters R.K., Martin J., Mattheisen M., Als T.D., Agerbo E., Baldursson G., Belliveau R., Bybjerg-Grauholm J., Bækvad-Hansen M., Cerrato F., Chambert K., Churchhouse C., Dumont A., Eriksson N., Gandal M., Goldstein J.I., Grasby K.L., Grove J., Neale B.M. (2019). Discovery of the first genome-wide significant risk loci for attention deficit/hyperactivity disorder. Nat. Genet..

[b0030] Desikan R.S., Ségonne F., Fischl B., Quinn B.T., Dickerson B.C., Blacker D., Buckner R.L., Dale A.M., Maguire R.P., Hyman B.T., Albert M.S., Killiany R.J. (2006). An automated labeling system for subdividing the human cerebral cortex on MRI scans into gyral based regions of interest. Neuroimage.

[b0035] Ding M., Zhang S., Zhu Z., Cai R., Fang J., Zhou C., Zhang X., Fang X. (2024). Influencing factors of different metabolic status in hospitalized patients with schizophrenia. Front. Psych..

[b0040] Dong K., Wang S., Qu C., Zheng K., Sun P. (2024). Schizophrenia and type 2 diabetes risk: a systematic review and meta-analysis. Front. Endocrinol..

[b0045] Durnin B.Y.J.V.G., Womersley J. (1973). Body fat assessed from total body density and its estimation from skinfold thickness: measurements on 481 men and women aged from 16 to 72 years. Br. J. Nutr..

[b0050] Elliott L.T., Sharp K., Alfaro-Almagro F., Shi S., Miller K.L., Douaud G., Marchini J., Smith S.M. (2018). Genome-wide association studies of brain imaging phenotypes in UK Biobank. Nature.

[b0055] Gurholt T.P., Kaufmann T., Frei O., Alnæs D., Haukvik U.K., van der Meer D., Moberget T., O'Connell K.S., Leinhard O.D., Linge J., Simon R., Smeland O.B., Sønderby I.E., Winterton A., Steen N.E., Westlye L.T., Andreassen O.A. (2021). Population-based body-brain mapping links brain morphology with anthropometrics and body composition. Transl. Psychiatry.

[b0060] Hagenaars S.P., Harris S.E., Davies G., Hill W.D., Liewald D.C.M., Ritchie S.J., Marioni R.E., Fawns-Ritchie C., Cullen B., Malik R., Worrall B.B., Sudlow C.L.M., Wardlaw J.M., Gallacher J., Pell J., McIntosh A.M., Smith D.J., Gale C.R., Deary I.J. (2016). Shared genetic aetiology between cognitive functions and physical and mental health in UK Biobank (N=112 151) and 24 GWAS consortia. Mol. Psychiatry.

[b0065] Hamzehpour L., Bohn T., Jaspers L., Grimm O. (2023). Exploring the link between functional connectivity of ventral tegmental area and physical fitness in schizophrenia and healthy controls. Eur. Neuropsychopharmacol..

[b0070] Hamzehpour L., Bohn T., Dutsch V., Jaspers L., Grimm O. (2024). From brain to body: exploring the connection between altered reward processing and physical fitness in schizophrenia. Psychiatry Res..

[b0075] Henderson D.C., Fan X., Sharma B., Copeland P.M., Borba C.P., Freudenreich O., Cather C., Evins A.E., Goff D.C. (2009). Waist circumference is the best anthropometric predictor for insulin resistance in nondiabetic patients with schizophrenia treated with clozapine but not olanzapine. J. Psychiatr. Pract..

[b0080] Hoogman M., Muetzel R., Guimaraes J.P., Shumskaya E., Mennes M., Zwiers M.P., Jahanshad N., Sudre G., Wolfers T., Earl E.A., Soliva Vila J.C., Vives-Gilabert Y., Khadka S., Novotny S.E., Hartman C.A., Heslenfeld D.J., Schweren L.J.S., Ambrosino S., Oranje B., de Zeeuw P., Chaim-Avancini T.M., Rosa P.G.P., Zanetti M.V., Malpas C.B., Kohls G., von Polier G.G., Seitz J., Biederman J., Doyle A.E., Dale A.M., van Erp T.G.M., Epstein J.N., Jernigan T.L., Baur-Streubel R., Ziegler G.C., Zierhut K.C., Schrantee A., Høvik M.F., Lundervold A.J., Kelly C., McCarthy H., Skokauskas N., O'Gorman Tuura R.L., Calvo A., Lera-Miguel S., Nicolau R., Chantiluke K.C., Christakou A., Vance A., Cercignani M., Gabel M.C., Asherson P., Baumeister S., Brandeis D., Hohmann S., Bramati I.E., Tovar-Moll F., Fallgatter A.J., Kardatzki B., Schwarz L., Anikin A., Baranov A., Gogberashvili T., Kapilushniy D., Solovieva A., El Marroun H., White T., Karkashadze G., Namazova-Baranova L., Ethofer T., Mattos P., Banaschewski T., Coghill D., Plessen K.J., Kuntsi J., Mehta M.A., Paloyelis Y., Harrison N.A., Bellgrove M.A., Silk T.J., Cubillo A.I., Rubia K., Lazaro L., Brem S., Walitza S., Frodl T., Zentis M., Castellanos F.X., Yoncheva Y.N., Haavik J., Reneman L., Conzelmann A., Lesch K.P., Pauli P., Reif A., Tamm L., Konrad K., Oberwelland W.E., Busatto G.F., Louza M.R., Durston S., Hoekstra P.J., Oosterlaan J., Stevens M.C., Ramos-Quiroga J.A., Vilarroya O., Fair D.A., Nigg J.T., Thompson P.M., Buitelaar J.K., Faraone S.V., Shaw P., Tiemeier H., Bralten J., Franke B. (2019). Brain imaging of the cortex in ADHD: a coordinated analysis of large-scale clinical and population-based samples. Am. J. Psychiatry.

[b0085] Jonas K., Clouston S., Li K., Fochtmann L.J., Lencz T., Malhotra A.K., Cicero D., Perlman G., Bromet E.J., Kotov R. (2019). Apolipoprotein E-ε4 allele predicts escalation of psychotic symptoms in late adulthood. Schizophr. Res..

[b0090] Kochunov P., Ryan M.C., Yang Q., Hatch K.S., Zhu A., Thomopoulos S.I., Jahanshad N., Schmaal L., Thompson P.M., Chen S., Du X., Adhikari B.M., Bruce H., Hare S., Goldwaser E.L., Kvarta M.D., Nichols T.E., Hong L.E. (2021). Comparison of regional brain deficit patterns in common psychiatric and neurological disorders as revealed by big data. NeuroImage: Clin..

[b0095] Kochunov P., Ma Y., Hatch K.S., Jahanshad N., Thompson P.M., Adhikari B.M., Bruce H., Van der vaart A., Goldwaser E.L., Sotiras A., Kvarta M.D., Ma T., Chen S., Nichols T.E., Hong L.E. (2022). Brain-wide versus genome-wide vulnerability biomarkers for severe mental illnesses. Hum. Brain Mapp..

[b0100] Laursen T.M., Munk-Olsen T., Vestergaard M. (2012). Life expectancy and cardiovascular mortality in persons with schizophrenia. Curr. Opin. Psychiatry.

[b0105] Liampas I., Kyriakoulopoulou P., Siokas V., Tsiamaki E., Stamati P., Kefalopoulou Z., Chroni E., Dardiotis E. (2024). Apolipoprotein E gene in α-synucleinopathies: a narrative review. Int. J. Mol. Sci..

[b0110] Ma Y., Kvarta M.D., Adhikari B.M., Chiappelli J., Du X., van der Vaart A., Goldwaser E.L., Bruce H., Hatch K.S., Gao S., Summerfelt A., Jahanshad N., Thompson P.M., Nichols T.E., Hong L.E., Kochunov P. (2023). Association between brain similarity to severe mental illnesses and comorbid cerebral, physical, and cognitive impairments. Neuroimage.

[b0115] Marek S., Tervo-Clemmens B., Calabro F.J., Montez D.F., Kay B.P., Hatoum A.S., Donohue M.R., Foran W., Miller R.L., Hendrickson T.J., Malone S.M., Kandala S., Feczko E., Miranda-Dominguez O., Graham A.M., Earl E.A., Perrone A.J., Cordova M., Doyle O., Moore L.A., Dosenbach N.U.F. (2022). Reproducible brain-wide association studies require thousands of individuals. Nature.

[b0120] Martinelli A., Leone S., Zamparini M., Carnevale M., Caterson I.D., Fuller N.R., Calza S., de Girolamo G. (2025). Association of weight status and waist circumference with physical activity in people with schizophrenia spectrum disorders and healthy controls. Brain Behav Immun.

[b0125] Mizuki Y., Sakamoto S., Okahisa Y., Yada Y., Hashimoto N., Takaki M., Yamada N. (2021). Mechanisms underlying the comorbidity of schizophrenia and type 2 diabetes mellitus. Int. J. Neuropsychopharmacol..

[b0130] Niarchou M., Byrne E.M., Trzaskowski M., Sidorenko J., Kemper K.E., McGrath J.J., O’Donovan M.C., Owen M.J., Wray N.R. (2020). Genome-wide association study of dietary intake in the UK biobank study and its associations with schizophrenia and other traits. Transl. Psychiatry.

[b0135] Rajkumar A.P., Horsdal H.T., Wimberley T., Cohen D., Mors O., Børglum A.D., Gasse C. (2017). Endogenous and antipsychotic-related risks for diabetes mellitus in young people with schizophrenia: a Danish population-based cohort study. Am. J. Psychiatry.

[b0140] Sharpe J.K., Stedman T.J., Byrne N.M., Wishart C., Hills A.P. (2006). Energy expenditure and physical activity in clozapine use: Implications for weight management. Aust. N. Z. J. Psychiatry.

[b0145] Smith J., Griffiths L.A., Band M., Horne D. (2020). Cardiometabolic risk in first episode psychosis patients. Front. Endocrinol..

[b0150] So H.C., Chau K.L., Ao F.K., Mo C.H., Sham P.C. (2019). Exploring shared genetic bases and causal relationships of schizophrenia and bipolar disorder with 28 cardiovascular and metabolic traits. Psychol. Med..

[b0155] Stahl S.M., Mignon L., Meyer J.M. (2009). Which comes first: atypical antipsychotic treatment or cardiometabolic risk?. Acta Psychiatr. Scand..

[b0160] Stein F., Lemmer G., Schmitt S., Brosch K., Meller T., Fischer E., Kraus C., Lenhard L., Köhnlein B., Murata H., Bäcker A., Müller M., Franz M., Förster K., Meinert S., Enneking V., Koch K., Grotegerd D., Nagels A., Nenadić I., Dannlowski U., Kircher T., Krug A. (2020). Factor analyses of multidimensional symptoms in a large group of patients with major depressive disorder, bipolar disorder, schizoaffective disorder and schizophrenia. Schizophr. Res..

[b0165] Stone W.S., Phillips M.R., Yang L.H., Kegeles L.S., Susser E.S., Lieberman J.A. (2023). To support a revisit. Neurodegenerative.

[b0170] Strassnig M., Brar J.S., Ganguli R. (2011). Low cardiorespiratory fitness and physical functional capacity in obese patients with schizophrenia. Schizophr. Res..

[b0175] Strassnig M., Signorile J., Gonzalez C., Harvey P.D. (2014). Physical performance and disability in schizophrenia. Schizophrenia research. Cognition.

[b0180] Thakore J.H., Mann J.N., Vlahos I., Martin A., Reznek R. (2002). Increased visceral fat distribution in drug-naive and drug-free patients with schizophrenia. Int. J. Obes. (Lond).

[b0185] Thakore J.H. (2005). Metabolic syndrome and schizophrenia. Br. J. Psychiatry.

[b0190] Tuominen L., Armio R., Hansen J.Y., Walta M., Koutsouleris N., Laurikainen H., Salokangas R.K., Mišić B., Hietala J. (2025). Molecular, physiological and functional features underlying antipsychotic medication use related cortical thinning. Transl. Psychiatry.

[b0195] Vancampfort D., Stubbs B., Mitchell A.J., De Hert M., Wampers M., Ward P.B., Rosenbaum S., Correll C.U. (2015). Risk of metabolic syndrome and its components in people with schizophrenia and related psychotic disorders, bipolar disorder and major depressive disorder: a systematic review and meta-analysis. World Psych..

[b0200] Vancampfort D., Firth J., Schuch F.B., Rosenbaum S., Mugisha J., Hallgren M., Probst M., Ward P.B., Gaughran F., De Hert M., Carvalho A.F., Stubbs B. (2017). Sedentary behavior and physical activity levels in people with schizophrenia, bipolar disorder and major depressive disorder: a global systematic review and meta-analysis. World Psych..

[b0205] Walburg F.S., Van Meijel B., Hoekstra T., Kol J., Pape L.M., De Joode J.W., Van Tulder M., Adriaanse M. (2023). Effectiveness of a lifestyle intervention for people with a severe mental illness in Dutch outpatient mental health care: a randomized clinical trial. JAMA Psychiat..

[b0210] Wallwork R.S., Fortgang R., Hashimoto R., Weinberger D.R., Dickinson D. (2012). Searching for a consensus five-factor model of the positive and negative syndrome scale for schizophrenia. Schizophr. Res..

[b0215] Wingo T.S., Liu Y., Gerasimov E.S., Vattathil S.M., Wynne M.E., Liu J., Lori A., Faundez V., Bennett D.A., Seyfried N.T., Levey A.I., Wingo A.P. (2022). Shared mechanisms across the major psychiatric and neurodegenerative diseases. Nat. Commun..

[b0220] Yamada Y., Matsumoto M., Iijima K., Sumiyoshi T. (2019). Specificity and continuity of schizophrenia and bipolar disorder: relation to biomarkers. Curr. Pharm. Des..

[b0225] Zhang-James Y., Helminen E.C., Liu J., Busatto G.F., Calvo A., Cercignani M., Chaim-Avancini T.M., Gabel M.C., Harrison N.A., Lazaro L., Lera-Miguel S., Louza M.R., Nicolau R., Rosa P.G.P., Schulte-Rutte M., Zanetti M.V., Ambrosino S., Asherson P., Banaschewski T., Faraone S.V. (2021). Evidence for similar structural brain anomalies in youth and adult attention-deficit/hyperactivity disorder: a machine learning analysis. Transl. Psychiatry.

