# Supplementary material for: Transdiagnostic neuroanatomical risk in schizophrenia: integrating regional vulnerability indices with anthropometric and fitness-based markers of cardiometabolic health
Source: Neuroimage Clin. 2025 Oct 27;48:103897. doi: 10.1016/j.nicl.2025.103897 (PMC12603745; doi:10.1016/j.nicl.2025.103897)
Supplement: Supplementary Data 1 [file mmc1.docx]

**Supplementary Material**

# Results of post-hoc power analysis

| Table 1: Post-hoc power analysis of RVI results of final sample size | | |  |
| --- | --- | --- | --- |
|  | p-value  (FDR adjusted) | Cohen’s D (\|d\|) | Post-hoc Power |
| AD | 0.01 | 0.6 | 0.77 |
| ADHD | 0.5 | 0.15 | 0.1 |
| BD | 0.0004 | 0.89 | 0.98 |
| T2D | 0.0005 | 0.86 | 0.97 |
| High BP | 0.11 | 0.38 | 0.41 |
| MDD | 0.33 | 0.23 | 0.18 |
| Metabolic syndrome | 0.09 | 0.41 | 0.46 |
| PD | 0.007 | 0.66 | 0.85 |
| SSD | < 0.0001 | 1.08 | 1 |

Notes: sample size SZ = 42, HC = 43; power for small effect (d = 0.2) = 0.15, power for medium effect (d = 0.5) = 0.61, power for large effect (d  = 0.8) = 0.95; AD, Alzheimer’s Disease; ADHD, Attention Deficit/Hyperactivity Disorder; BD, Bipolar Disorder; T2D, Type 2 Diabetes; High BP, high blood pressure; MDD, major depressive disorder; PD, Parkinson’s Disease; SSD, schizophrenia spectrum disorder; RVI, Regional Vulnerability Index

# Post-hoc correlation results

| Table 2: Spearman correlation of chlorpromazine equivalent with significant RVI- and PCA results | | |
| --- | --- | --- |
|  | Correlation coefficient | p-value  (FDR-adjusted) |
| SSD-RVI | -0.03 | 0.94 |
| BD-RVI | 0.15 | 0.65 |
| AD-RVI | -0.21 | 0.46 |
| PD-RVI | -0.24 | 0.46 |
| T2D-RVI | 0.23 | 0.46 |
| PC2 | -0.12 | 0.65 |
| PC3 | 0.01 | 0.94 |

Notes: Spearman correlation between chlorpromazine equivalent and significant RVIs and PCs, controlling for sex and BMI; SSD-RVI, RVI for schizophrenia spectrum disorder; BD-RVI, RVI for bipolar disorder; AD-RVI, RVI for Alzheimer’s Disease; PD-RVI, RVI for Parkinson’s Disease; T2D-RVI. RVI for Type 2 Diabetes; PC2, principal component 2 “Physical Fitness Dimensions”; PC3, principal component “psychosis-spectrum vulnerability”

# Principal Component Analysis excluding variables below MSA 0.5

In order to support our decision to include the variables RVI Schizophrenia Spectrum Disorder and RVI Bipolar Disorder in our PCA, although the Kaiser-Meyer-Olkin test revealed a measure of sampling adequacy (MSA) below 0.5 for both variables, we performed another post-hoc PCA excluding them. The factor loadings and explained variances can be taken from Supplementary Table 3. To compare the loadings from both analyses we used the Tucker congruence coefficient (φ). The results indicate nearly identical values (φ(PC1) = 1; φ(PC2) = 1; φ(PC3) = 0.93), supporting the stability of the PCA despite the inclusion of the RVIs for Schizophrenia Spectrum Disorder and Bipolar Disorder.

| Table 3: PCA loadings excluding all variables below MSA 0.5 | | | | |
| --- | --- | --- | --- | --- |
|  | PC1 | PC2 | PC3 |  |
| PAS | 0.01 | -0.01 | 0.63 |  |
| VO_2_max | 0.05 | -0.72 | -0.02 |  |
| WHtR | 0.17 | 0.65 | 0.06 |  |
| skin folds sum | 0.06 | 0.88 | 0.13 |  |
| total body fat | 0.01 | 0.9 | 0.12 |  |
| jump width | 0.13 | -0.8 | -0.14 |  |
| handgrip strength | 0.1 | -0.5 | -0.32 |  |
| WHODAS | 0.25 | 0.21 | 0.67 |  |
| CDSS | -0.01 | 0.28 | 0.53 |  |
| ADHD-RVI | 0.85 | -0.05 | -0.28 |  |
| T2D-RVI | 0.67 | -0.04 | 0.47 |  |
| MDD-RVI | 0.93 | 0.04 | 0.05 |  |
| HighBP-RVI | 0.92 | 0.04 | -0.03 |  |
| MET-RVI | 0.94 | 0.02 | 0.12 |  |
| PD-RVI | -0.42 | 0.12 | 0.57 |  |
| explained variance in % | 27 | 23,8 | 12,8 |  |

Notes: MSA, measure of sampling adequacy (after Kaiser-Meyer-Olkin test); PAS, Physical Anhedonia Scales; VO2max, maximal oxygen capacity; WHtR, waist-to-height ratio; WHODAS, World Health Organization Disability Assessment Schedule; CDSS, Calgary Depression Scale for Schizophrenia; ADHD-RVI, RVI for Attention Deficit/Hyperactivity Disorder; T2D-RVI, RVI for Type 2 Diabetes; MDD-RVI, RVI for major depressive disorder; HighBP-RVI, RVI for hypertension; MET-RVI, RVI for metabolic syndrome; PD-RVI, RVI for Parkinson’s Disease
